# Rapid construction of genome map for large yellow croaker (*Larimichthys crocea*) by the whole-genome mapping in BioNano Genomics Irys system

**DOI:** 10.1186/s12864-015-1871-z

**Published:** 2015-09-03

**Authors:** Shijun Xiao, Jiongtang Li, Fengshou Ma, Lujing Fang, Shuangbin Xu, Wei Chen, Zhi Yong Wang

**Affiliations:** Key Laboratory of Healthy Mariculture in the East China Sea, Ministry of Agriculture; Fisheries College, Jimei University, Yindou Road, Xiamen, P.R. China; Chinese Academy of Fishery Sciences, Yongding Road, Beijing, P.R. China; Genergy Inc., Shanghai, P.R. China

**Keywords:** Large yellow croaker, Gene map, Whole-genome mapping, Genome assembly

## Abstract

**Background:**

Large yellow croaker (*Larimichthys crocea*) is an important commercial fish in China and East-Asia. The annual product of the species from the aqua-farming industry is about 90 thousand tons. In spite of its economic importance, genetic studies of economic traits and genomic selections of the species are hindered by the lack of genomic resources. Specifically, a whole-genome physical map of large yellow croaker is still missing. The traditional BAC-based fingerprint method is extremely time- and labour-consuming. Here we report the first genome map construction using the high-throughput whole-genome mapping technique by nanochannel arrays in BioNano Genomics Irys system.

**Results:**

For an optimal marker density of ~10 per 100 kb, the nicking endonuclease Nt.BspQ1 was chosen for the genome map generation. 645,305 DNA molecules with a total length of ~112 Gb were labelled and detected, covering more than 160X of the large yellow croaker genome. Employing IrysView package and signature patterns in raw DNA molecules, a whole-genome map of large yellow croaker was assembled into 686 maps with a total length of 727 Mb, which was consistent with the estimated genome size. The N50 length of the whole-genome map, including 126 maps, was up to 1.7 Mb. The excellent hybrid alignment with large yellow croaker draft genome validated the consensus genome map assembly and highlighted a promising application of whole-genome mapping on draft genome sequence super-scaffolding. The genome map data of large yellow croaker are accessible on lycgenomics.jmu.edu.cn/pm.

**Conclusion:**

Using the state-of-the-art whole-genome mapping technique in Irys system, the first whole-genome map for large yellow croaker has been constructed and thus highly facilitates the ongoing genomic and evolutionary studies for the species. To our knowledge, this is the first public report on genome map construction by the whole-genome mapping for aquatic-organisms. Our study demonstrates a promising application of the whole-genome mapping on genome maps construction for other non-model organisms in a fast and reliable manner.

**Electronic supplementary material:**

The online version of this article (doi:10.1186/s12864-015-1871-z) contains supplementary material, which is available to authorized users.

## Background

Large yellow croaker (*Larimichthys crocea*), belonging to the family of Osteichthyes and the order of Perciformes, is an economic fish in China and East Asia [1]. With an annual product of 90 thousand ton, the species is one of the most important mariculture fish in China [2]. Nowadays, the direct annual marker value of large yellow croaker is about 60 thousand dollar. In the last decades, the wild population of the species has collapsed due to over-fishing and habitat degeneration [3, 4]. Both the environmental changes and over-dense aquaculture pose enormous challenges on economic trait degeneration, stress adaption and susceptibility to infectious disease for large yellow croaker [1]. Therefore, it is an urgent requisite to explore the association analysis of functional genes with aquaculture performance in order to breed new large yellow croaker strain with better economic traits and higher anti-infection to meet the requirement of the modern aquaculture industry [2]. Many literatures have been published for gene functions [5, 6], mitochondrial genome [7] and transcriptome analysis [8] for large yellow croaker in the last few years. Genetic maps of the large yellow croaker has also been reported using amplified fragment length polymorphism (AFLP) and microsatellites markers aiming at pseudo-chromosome construction and functional gene identification [9, 10]. Recently, scientists have reported the draft genomes of large yellow croaker in 2014 and 2015 (referred as 2014draft and 2015draft hereafter, respectively) [11, 12], which highly facilitate the functional gene mapping and genome-wide association studies (GWAS). In spite of these efforts in genomic analysis of large yellow croaker, a physical map for the species is still lacking, which hinders the fine genomic sequence assembly and further investigation of complex traits by multi-omics analysis.

The advent and development of Next Generation Sequencing (NGS) have already brought scientific studies in many fields including agriculture and medicine into an era of genomics in the last decade [13, 14]. Nowadays, it is possible to *de novo* assembly a draft genome of a species in several months [15]. However, the genome assembly from short NGS reads remains a challenge [16], especially for aquatic organisms with high repeat context and complex genomic heterozygosity. Physical mapping is one prevalent technique that used to facilitate sequence assembly and functional gene location for genomic projects of many species [17–19]. It employs specific fingerprints along DNA molecules and, more importantly, can span large repeat and heterozygous regions; thus the signature patterns in physical maps provide valuable framework for fine genomic sequence assembly. Traditional BAC-based physical mapping technologies have been used in many aquatic fish species, including Nile tilapia (*Oreochromis niloticus*) [20], Atlantic salmon(*Salmo salar*) [21] channel catfish (*Ictalurus punctatus*) [22], Asian sea bass (*Lates calcarifer*) [23], rainbow trout (*Oncorhynchus mykiss*) [24] and half-smooth tongue sole (*Cynoglossus semilaevis*) [25]. Yet, BAC-based fingerprint experiments are extremely time consuming and labour costly. In the last few years, scientists have invented high-throughput methods to identify restraint sites of mega-sized DNA molecules directly, leading to mainly two novel physical mapping technologies: optical mapping [26] and whole-genome mapping [27–29]. In optical mapping, large DNA molecules are fixed and digested on polylysine-treated glass surfaces and DNA fragments and digestions are visualized by fluorescence microscopy, so that ordered restriction maps of those DNA molecules are derived from digital images [26, 30–32]. The advent of optical mapping has led to many successful applications in micro-organism chromosome map constructions [31–33]. However, the non-linear fixation of DNA molecules on glass surfaces and completed sequence digestion influence native molecular stressed conformations and pose tremendous challenges for the accurate estimation of signature length in DNA fragments. To overcome those problems, scientists have recently invented the whole-genome mapping by utilizing nanochannel arrays and nicking endonuclease to minimize the bias for the nicking site detection [34, 35]. Equipped with this state-of-the-art technology, BioNano Genomics Irys system is developed and could linearize a single DNA molecule in one 45 × 45 nm nanochannel with buffer to detect DNA molecule length and locations of nicking sites [34]. In the last few years, Irys system has been used to build genome maps as well as to guide high-confidence assembly of extra-ordinary complex genomic regions in *aegilops tauschii* [36] and in human [37]. Given such advantages, the genome map application of the whole-genome mapping in Irys system for non-model organisms is rather promising; however, to our best knowledge, a public report of genome map construction applying Irys system for non-model organisms has not been published so far.

Here in this study, we employed Irys system to construct a whole-genome map of large yellow croaker. A large amount (~120 Gb) of DNA fragment information were detected from the system. The high coverage (~160X) of raw data assured sufficient DNA molecules for genome map assembly. The genome maps were validated by the alignment with draft genome sequences. We believe the constructed whole-genome map for large yellow croaker can provide a valuable framework for super-scaffolding draft genome sequences, building pseudo-chromosomes and facilitating the following integration analysis with genetic maps.

## Results and discussions

### Nicking endonuclease selection

Four candidate enzymes in Table 1, Nt.BspQ1, Nb.Bsml, Nb.BbvCI and Nb.BsrD1, were provided for the whole-genome mapping in Irys system. To select a restriction endonuclease enzyme with a proper label density for the whole-genome mapping, the public draft genome 2014draft [11] of large yellow croaker was used to estimate the endonuclease site density. As shown in Table 1, Nb.BsrD1 and Nt.BspQ1 exhibited the highest (41.1) and lowest (13.7) site density, respectively. Based on previously estimated resolution for the platform, the experimental label densities were evaluated *in silico* by omitting nicking sites within 500 bp to each other; therefore the label densities were smaller than nick site densities because of the limited resolution between nick sites. To satisfy the optimal label density of ~10 per 100 kb in Irys system, we selected the nicking endonuclease of Nt.BspQ1 with the specific motif of GCTCTTC for the whole-genome mapping (Table 1). According to the empirical performances in other species, the whole genome average label density may fluctuate by 15 % around the pilot estimation, suggesting a reasonable average label density of Nt.BspQ1 nicking sites for large yellow croaker genome should fall into the range of 9.35 ~ 12.85 per 100 kb.

**Table 1:** Label density estimation. Label density estimation for four restriction endonuclease enzyme candidates

Enzyme	Nick density (nicks per 100 kb)	Label density (labels per 100 kb)
Nt.BspQ1	13.7	11.0
Nb.Bsml	35.9	22.6
Nb.BbvCI	17.6	13.4
Nb.BsrD1	41.1	24.4

### DNA molecules and labels

**Fig. 1:**
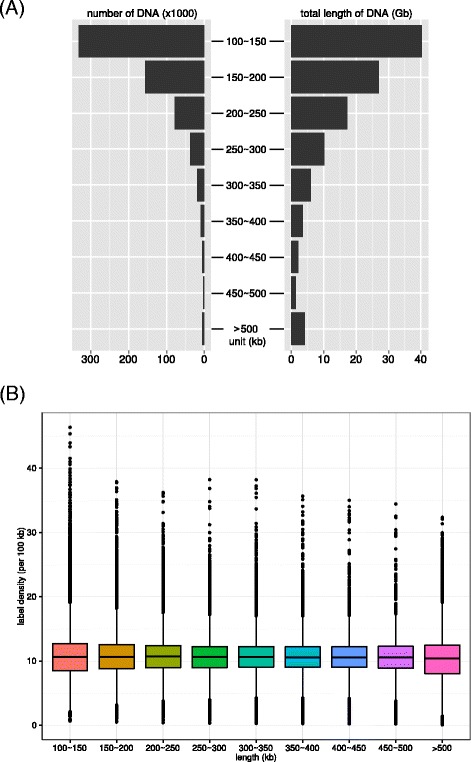
The number, total length and label density distribution of raw DNA molecules from whole-genomic mapping. **a** Sequence number (left) and total length (right) of raw DNA molecules for each length interval; **b** Label density in boxplot for each length interval. The estimated overall average label density was ~10.6

DNA lengths and nicking sites information were recorded as digital images in Irys system (see detail in Material and Method). From recognition processes of the resulted digital images, we obtained 645,305 valid DNA molecules longer than 100 kb. The raw DNA molecules number and length distribution were depicted in Fig. 1a. The N50 length of all DNA fragments was ~176 kb. Notably, the longest DNA molecule was 2.88 Mb, which was even comparable to the longest assembled physical map in previous literatures. Mega-sized DNA molecules resulted from long DNA library construction and nanochannel design [28] in Irys system would extremely improve the genome map assembly. The total throughput of raw DNA molecules was about 112 Gb, covering about 160X of large yellow croaker genome. The high-throughput of raw DNA molecules suggested that Irys system outperformed previous reported BAC-based fingerprint method on raw data output [19–25, 38] and provided sufficient DNA fragments for a reliable whole-genome map assembly. We noted that almost half of DNA lengths were catalogued as 100 ~ 150 kb but those molecules only account for 35 % (~40 Gb) of the overall throughput, implying longer DNA molecules contribute significantly to the total data. A plenty of DNA molecules (5,758) were longer than 500 kb and the total length of those molecules was ~4.1 Gb, covering 5.5X of large yellow croaker genome (Fig. 1a). We listed the detailed DNA molecule numbers and average depths information against various length thresholds in Additional file 1: Table S1. The average depth was still larger than 100X even with a length threshold as long as 150 kb.

To validate the label density within DNA molecules, we illuminated the average label density against DNA length in Fig. 1b. The label density of Nt.BspQ1 was rather stable as ~10.6 in all length categories, which excellently agreed with our previous pilot theoretical evaluation. However, we found that the label densities in one DNA molecule varied from 0.08 to 46.4, implying the heterogeneous nicking site distribution along large yellow croaker genome. To evaluate signature patterns of raw DNA molecules and to estimate accuracy of motif labelling by Irys system, we aligned those signature patterns in DNA molecules longer than 150 kb to the draft genomes of large yellow croaker (see Material and Method for detail). As a result, ~60 and ~67 % of raw DNA molecules were successfully thoroughly mapped upon 2014draft and 2015draft genome sequences, respectively. The alignment ratio was reasonable for three main reasons: firstly, the draft genome sequences were not completed. Because of length scale of DNA molecules (>100 kb), the incompleteness of draft genome would influence the raw data mapping, especially for those DNA molecules locating at sequence ends or in unassembled genome sequences; secondly, the whole-genome mapping and *de novo* sequencing experiments were carried out with different individuals, therefore inherent genetic diversifications unavoidably raised the divergence for alignments; thirdly, false positives (false present markers in DNA molecules) and false negatives (true markers absent in DNA molecules) caused by enzymatic activities, limited labelling resolution, camera recording and software processing also mislead DNA mapping. Our analysis also implied that the bioinformatics utilities (IrysView packages) had much room for further improvement for more efficient genome map applications. Based on mis-matches observed in alignments between raw DNA molecules and genome sequences, we estimated the average false positive to genome sequences in this experiment to be 1.5 per 100 kb, which was close to the estimation of 1 per 100 kb in the whole-genome mapping for *E. coli*. Note that inherent sample-wise genetic diversifications of individuals may also contribute to the false positive rate estimation; thereby the technical false positive rate should be lower. Meanwhile, we calculated the average Bases per pixel (BPP) in the labelling to be 504 bp, implying the technique exhibited an excellent resolution to detect fluorescence labels ~500 bp around real nicking sites in the experiment. Based on above analyses, we concluded that the whole-genome mapping in Irys system provided high throughput unbiased raw DNA molecules with a proper resolution for large yellow croaker genome map construction.

### Whole-genome map assembly from DNA molecules

**Fig. 2:**
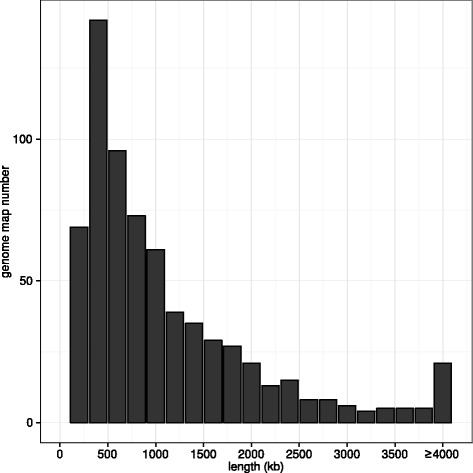
The length distribution of genome maps for large yellow croaker. Note that all maps longer than 4 Mb were accumulated in the last bar

To build a whole-genome map, raw DNA molecules longer than 100 kb were used in Assembler utility of IrysView package to construct consensus genome maps of large yellow croaker. As a result, 686 genome maps were assembled with a total length of 727 Mb, which was excellently consistent with the genome size estimation of 725 ± 14.6 Mb in flow cytometry experiments [39]. Additional file 1: Figure S1 illuminated an example of consensus map construction from raw DNA molecules. As shown in Table 2, the N50 length of the assembled genome maps for large yellow croaker genome was ~1.7 Mb, with the longest and smallest map to be 105 kb and 9 Mb, respectively. The average length of genome maps was ~1.06 Mb (Fig. 2). We observed that the N50 length was significantly longer than the mean length of genome maps, implying a quality whole-genome map assembly. Among all genome maps, 94 of them (~14 %) were longer than 2 Mb, representing 305 Mb (~42 %) of the whole large yellow croaker genome.

**Table 2:** Genome map and draft genome sequence statistics. Basis statistics for raw DNA molecules, assembled genome maps and published draft genome sequences

	Raw DNA molecules	Genome maps	2014draft [11]	2015draft [12]
Number	645,305	686	3,478	6,013
Total length (Mb)	111,830	727	644	679
N50 (kb)	176	1,714	498	1,030
min (kb)	100	105	2.0	0.5
max (kb)	2,880	9,002	3,825	4,914

**Fig. 3:**
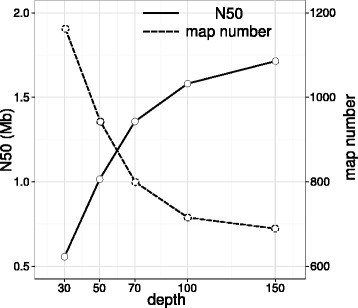
N50 and map number of the whole-genome map assembly under different molecule depth. We randomly sampled subsets from total raw DNA molecules to assembly genome map, aiming to estimate how molecule depth influenced N50 length (solid line; left Y-axis) and map number (dashed line; right Y-axis). All parameters were identical for all map assembly

To investigate how DNA molecule depth influenced genome map assembly and to determine the minimal data required for a proper whole-genome mapping of large yellow croaker, subsets of DNA fragments with various depths were sampled from total raw DNA molecules. Figure 3 showed that N50 length of genome maps exceeded 1 Mb with a molecule depth of 50X and increased almost linearly by improving molecule depth from 30X to 70X, while the corresponding total map number decreased sharply from 1162 (30X) to 799 (70X). Although a slightly weakening tendency was observed with molecule depth from 70X to 100X, both map number (715) and N50 length (~1.58 Mb) were significantly improved. We found that the map number and N50 length under the depth of 100X have already surpassed draft genome sequences of 2014 draft and 2015 draft for large yellow croaker (Table 2). The further accumulation of molecule depth larger than 100X led to relatively smaller changes of map number and N50 length. Considering the trade-off between experimental cost and proper genome map assembly, a molecule depth of ~100X may represent an optimal molecule depth with the best cost/performance ratio for the whole-genome mapping of large yellow croaker. This analysis offered a valuable reference for the following projects using the whole-genome mapping technique to construct genome maps for other non-model organisms, but the caution should always be taken that the optimal molecule depth might vary with genomic complexity in organisms.

### Genome map evaluation

Contrast with previous reports based on BAC-end fingerprint, the whole-genome map in this work was constructed directly from mega-sized genomic DNA molecules; therefore it was difficult to evaluate genome maps *via* PCR amplifications by primers designed from BAC clones. However, the length scale of consensus maps enables us to directly compare all published genome sequences with the whole-genome map. The underlying philosophy was that if genome maps were correctly assembled, there should be a large proportion of alignments between genome sequences and maps. We believe that the hybrid alignment of genome sequences and map is extremely essential because the analysis not only evaluates the accuracy of genome maps but also answer the question that to what extent can the whole-genome map facilitate the genome sequence super-scaffolding.

**Fig. 4:**
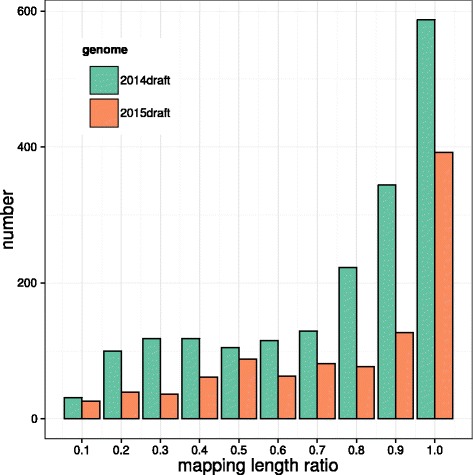
Mapping length ratio distribution for the alignment of genome sequences upon genome maps. The mapping length ratio, defined as map length divided by total length, were estimated for reference genome 2014draft [11] (green) and 2015draft [12] (orange). Only mappings with the highest confidence score of each genomic sequence were collected for the analysis. Note that small mapping length ratios may represent large scale nicking site heterozygosity or mappings at map ends

Since the published genome sequences of large yellow croaker were remarkably more fragmented than the assembled genome maps (Table 2), we aligned all genome sequences to the whole-genome map by RefAligner utility in IrysView package (see detail in Materials and methods). To improve the accuracy of the hybrid alignment, only genome sequences with more than 6 nicking sites, leaving 1,870 fragments, were used in the analysis. As shown in Fig. 4, we found that 1,154 (~62 %) and 931 (~50 %) genomic sequences aligned upon genome maps with a mapping length ratio higher than 0.7 and 0.8 for 2014draft, respectively. More than 300 genome sequences were thoroughly aligned to genome maps (mapping length ratio ≥ 0.95). The excellent alignments were also observed for genome sequences from 2015draft (Fig. 4). Considering the fact that genome sequences of large yellow croaker were also fragmented, our alignment results confirmed the reliability of the whole-genome map constructed from raw DNA molecules.

**Fig. 5:**
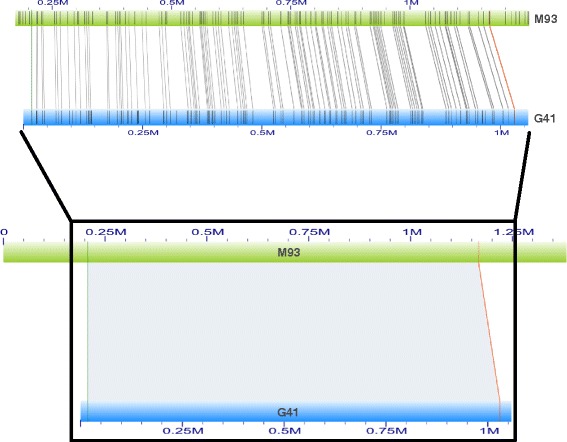
The detail nicking site alignment of genome sequences to genome maps. The genome sequence and genome map were shown in light blue and green colour and the corresponding names begun with G and M, respectively. The zoom in inset illuminated the detail nicking site alignments. The light green and red boundaries represented the beginning and ending sites for the alignment

**Fig. 6:**
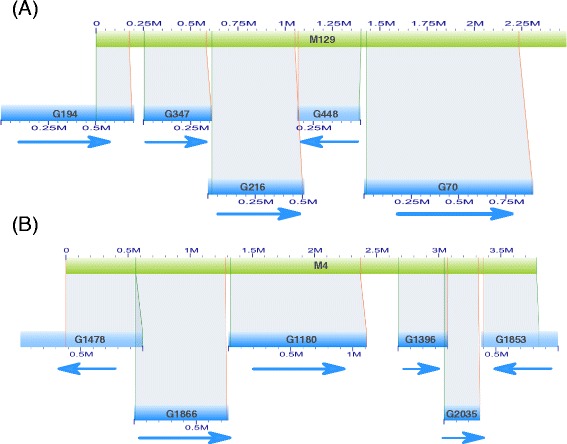
The validation of genome map by multi-alignments from genome sequences. Two hybrid alignment examples were illuminated for M129 of 2014 draft (**a**) and M4 of 2015 draft (**b**). The colour and name scheme in genome map and genome sequences was identical with those in Fig. 5. The gaps between genome sequences may represent un-sampled or low-complexity regions in genomic NGS sequencing. The arrows beneath genome sequences showed the mapping direction along genome maps

To visualize nicking site alignments between genome sequences and maps, Fig. 5 illuminated the detail schema of local alignment of 2014draft genome sequence 41 (G41) upon genome map 93 (M93). The excellent accordance of nicking sites spanning ~1 Mb region confirmed the accuracy of the whole-genome mapping. More notably, we found that many genome maps were thoroughly aligned by shorter reference genome sequences. Examples were demonstrated in Fig. 6a, b that genome map M129 and M4 were covered by two genome sequence groups of G149, G347, G216, G448 and G70 from 2014draft and G1478, G1866, G1180, G1396, G2035 and G1853 from 2015draft, respectively. The gaps between adjacent alignment boundaries likely represent regions that failed to cover in genomic NGS sequencing projects or low-complexity sequences. In addition, the mapping directions were also observed in those hybrid alignments; for example 2014draft G448 was found to reversely aligned to M129 (Fig. 6a). Given the multi-alignment information on genome maps, orders of genome sequences along chromosomes, as well as their directions, were clearly assigned. The alignment analysis of genome sequences upon the whole-genome map demonstrated that genome maps successfully strode gapped regions in genomic NGS sequencing experiments and thus served as an ideal guidance for the following genome sequences super-scaffolding and integrative analysis with genetic maps.

## Conclusions

Large yellow croaker is an important commercial fish in China and East Asia, however, genomic resources of the species, especially for physical maps, are still lacking, hindering the following genomic information integration and functional gene mapping. The construction of whole-genome physical map could facilitate genome sequence assembly and map-based gene identification [25, 40]. Therefore, a completed and accurate physical map of large yellow croaker could provide valuable reference for genomic studies of this important species.

The traditional physical map construction method based on BAC libraries and restriction fingerprinting is extremely time- and labour-expensive. More importantly, those libraries barely cover 100 % of the genome, because there would be some missing genomic regions caused by restriction enzyme bias, leaving gaps in the assembled physical map [25]. Employing nano- and optical-technologies, Irys system enables researchers to perform the whole-genome mapping in a high-throughput manner by nicking endonuclease in mega-sized DNA molecules directly [27]. The system has been applied in high-resolution genome map construction for complex regions of several model species, such as human and wheat [34–36]. In this work, we employed the state-of-the-art technique to probe signature patterns in mega-sized DNA molecules and assembled the first whole-genome map of large yellow croaker. With GCTCTTC motif recognized by the nicking endonuclease of Nt.BspQ1, signature patterns in 645,305 DNA molecules (≥100 kb) were detected from the system and assembled into a whole-genome map of large yellow croaker, resulting into 686 maps with a N50 length of ~1.7 Mb. The total length of genome maps was 727 Mb, agreeing well with the flow cytometry estimation [39]. The influence of DNA molecule depth on map assembly were investigated and discussed. The hybrid alignment between constructed genome maps and draft genome sequences validated the map assembly and illuminated the potential application of the whole-genome mapping for draft genome sequences super-scaffolding.

With the completed and reliable map resources, the comprehensive integration analysis of genetic map and genome map could highly improve the genomic sequence assembly, evolutionary investigations and genomic selections of large yellow croaker. Meanwhile, this is the first public report on genome map construction for none-model organisms by Irys system. Our studies have validated the whole-genome mapping technique and provided valuable reference for rapid genome map construction of other organisms.

## Methods

### Ethics statement

This study was approved by the Animal Care and Use committee of Fisheries College of Jimei University.

### Sample preparation and mega-sized DNA extraction

Large yellow croaker samples were obtained from the breeding base of Jimei University in Ningde, Fujian, China. Venous blood samples from fish tails were collected for genome DNA extraction. To extract long genomic DNA molecules from blood samples, peripheral mononuclear cells (PBMCs) from whole blood were isolated after density gradient centrifugation with Ficoll-Paque PLUS medium and BioNano’s commercial plug lysis protocol. The megabase-sized DNA molecules were then extracted according to IrysPrep TM Plug Lysis Long DNA Isolation protocol. Qubit 2.0 was used to quantify DNA concentration in solution. DNA samples met the following criterions were used for further experiments: 1) the concentration of DNA ranged from 35 *ng/μL* to 200 *ng/μL*; 2) the coefficient of variation (CV) from top, middle, and bottom of the DNA solution was less than 25 %; 3) contained mega base size DNA as measured by pulsed field gel electrophoresis (PFGE).

### DNA molecules labelling and data collection

After the extraction and quality control of mega-sized DNA molecules, sequence specific labelling of DNA molecules were performed by Nick, Labelling, Repair and Staining steps according to IrysPrep TM NLRS assay (900 ng): sequence specificity were provided by the Nickase, Nt.BspQ1; labelling was carried out by a nick translation process in the presence of a fluorophore-labelled nucleotide; the labelled nicks were repaired to restore strand integrity; DNA molecules were stained for the backbone visualization. Automated by the Irys system, stained DNA molecules were loaded into BioNano Genomics nanochannel chips by electrophoresis. Twelve volts were applied to concentrate DNA molecules into the port, thirty volts were used to unwound DNA molecules within the pillar structures and to move long molecules into nanochannels with buffers, and ten volts was applied to linearize DNA molecules in 45 × 45 nm nanochannels. Label positions and lengths of DNA molecules were recorded by the on-board CCD camera using green and blue lasers in the BioNano Genomics Irys system.

### Data conversion and bioinformatics processes

Digital images illuminating nick sites in raw DNA molecules from BioNano Genomics Irys system were processed by IrysView package to obtain basic labelling and DNA length information. The signature patterns in DNA molecules were then clustered and assembled by Assembler in IrysView package as described in previous publication [34]. Briefly, DNA molecules were firstly clustered by label distribution with the R package of fastcluster [41], then label positions along DNA molecules in one cluster were fitted by Gaussian functions. The final assembled maps were constructed by identified peaks in the fitted curves.

For the hybrid alignment of draft genome and genome maps, genome sequences were “digested” into signature patterns according to the restraint site of Nt.BspQ1 by knicker software (http://www.bnxinstall.com/knickers/Knickers.htm). The alignment with genome maps was performed with RefAligner utility in IrysView package. The parameters for the average false positive (FP) and false negative (FN) were set to 1.5 and 0.15 (−FP 1.5 -FN 0.15), respectively. The resulted alignment information, including confidence score [42], was used for the following statistics, such as mapping length ratio calculation (alignment length to the fragment length). Because of the flanking regions before the first nicking site and after the last nicking site, we defined a thorough alignment if mapping length ratio ≥ 0.95. The visualization of the hybrid alignment between genome maps and sequences were performed and snapshot in IrysView.

## Availability of supporting data

The genome map data of large yellow croaker are accessible on lycgenomics.jmu.edu.cn/pm. Raw DNA molecules cmap files are available in additional files.

## Additional file

Additional file 1: Table S1. DNA molecule statistics with various length filtering. **Figure S1.** The schematic illustration of the genome map assembly from DNA molecules. (DOCX 429 kb)

## Abbreviations

AFLP: Amplified fragment length polymorphism
GWAS: Genome-wide association studies
NGS: Next Generation Sequencing
BPP: Bases per pixel
PMBCs: Peripheral mononuclear cells
CV: Coefficient of variation
PFGE: Pulsed field gel electrophoresis
FP: False positive
FN: False negative
