# Supplementary material for: Rapid construction of genome map for large yellow croaker (Larimichthys crocea) by the whole-genome mapping in BioNano Genomics Irys system
Source: BMC Genomics. 2015 Sep 3;16(1):670. doi: 10.1186/s12864-015-1871-z (PMC4559010; doi:10.1186/s12864-015-1871-z)

# Supplementary Information for

# Rapid construction of genome map for large yellow croaker (*Larimichthys crocea*) by the whole-genome mapping in BioNano Genomics Irys system

Shijun Xiao^1^, Jiongtang Li^2^, Fengshou Ma^3^, Lujing Fang^1^, Shuangbin Xu^1^, Wei Chen^1^ and Zhi Yong Wang^1,*^

1 Fisheries College, Jimei University, Yindou Road, Xiamen, P.R. China

2 Chinese Academy of Fishery Sciences, Yongding Road, Beijing, P.R. China

3 Genergy Inc., Shanghai, P.R. China

Email: Zhiyong Wang - [zywang@jmu.edu.cn](mailto:zywang@jmu.edu.cn);

# * Corresponding author

**Supplementary Table 1: DNA molecule statistics with various length filtering.** DNA molecule number, total length and genome coverage depth are listed against various raw DNA length filtering thresholds.

| Length threshold (kb) | number | total length (Mb) | depth (X) |
| --- | --- | --- | --- |
| 100 | 645,305 | 111,830 | 160 |
| 105 | 596,421 | 106,819 | 153 |
| 110 | 554,509 | 102,310 | 146 |
| 115 | 516,112 | 97,993 | 140 |
| 120 | 478,014 | 93,519 | 134 |
| 125 | 445,540 | 89,542 | 128 |
| 130 | 413,695 | 85,482 | 122 |
| 135 | 385,497 | 81,745 | 117 |
| 140 | 360,082 | 78,250 | 112 |
| 145 | 335,161 | 74,699 | 107 |
| 150 | 313,552 | 71,512 | 102 |

**Supplementary Figure 1: The schematic illustration of the genome map assembly from DNA molecules.**

The top light blue bar with length marks represents the assembled genome map (~4.2 Mb) and the below blue sticks shows raw DNA molecules contributing to the assembly. The dark blue lines in the genome map and dots in raw DNA molecules are restriction endonuclease sites, identifying signature patterns in those sequences.


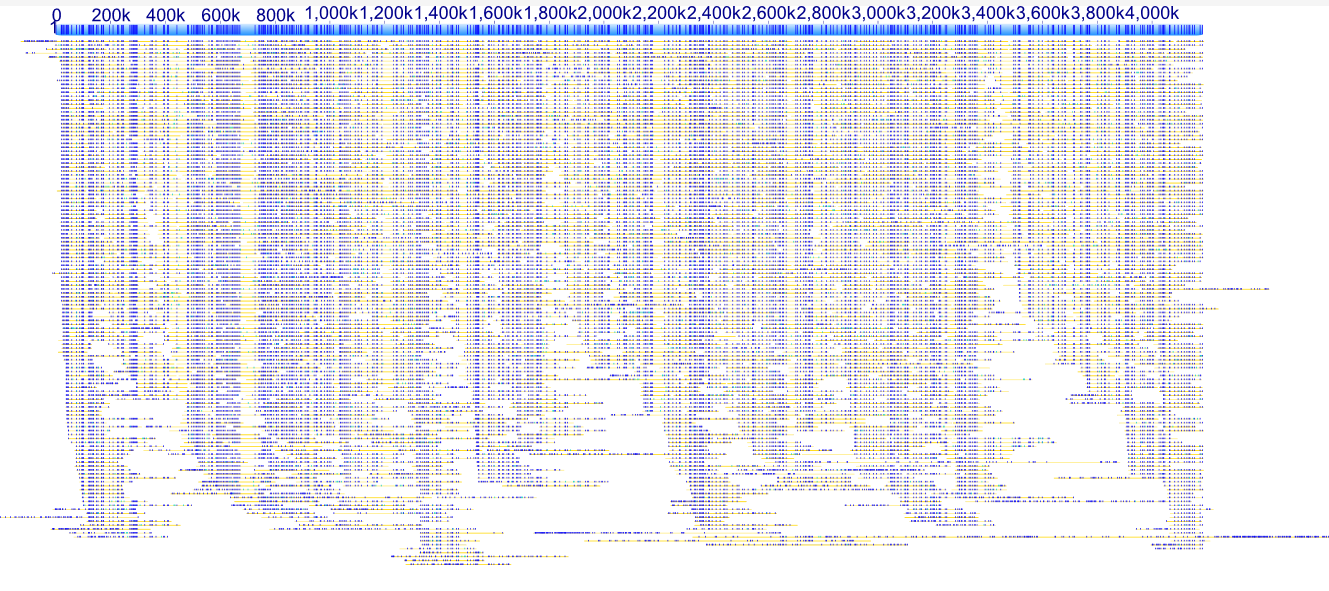

Supplement: Additional file 1: Table S1. — DNA molecule statistics with various length filtering. Figure S1. The schematic illustration of the genome map assembly from DNA molecules. (DOCX 429 kb) [file 12864_2015_1871_MOESM1_ESM.docx]
